# Self-compassion and self-efficacy as biopsychosocial resources in healthy aging: Associations with pain, health behaviors, postural awareness, and quality of life

**DOI:** 10.1371/journal.pone.0356869

**Published:** 2026-09-16

**Authors:** Nuray Alaca, Sılasu Turhan, Ali Ömer Acar, Sergen Öztürk

**Affiliations:** 1 Department of Physiotherapy and Rehabilitation, Faculty of Health Sciences, Acibadem Mehmet Ali Aydinlar University, Istanbul, Turkey; 2 Department of Physiotherapy and Rehabilitation, Graduate School of Health Sciences, Acibadem Mehmet Ali Aydinlar University, Istanbul, Turkey; 3 Physiotherapy and Rehabilitation PhD Program, The Graduate Education Institute, Istinye University, Istanbul, Turkey; 4 Physiotherapy and Rehabilitation PhD Program, The Graduate Education Institute, Biruni University, Istanbul, Turkey; 5 Physiotherapy and Rehabilitation PhD Program, Institute of Health Sciences, Marmara, University, Istanbul, Turkey; TIU: Tishk International University, IRAQ

## Abstract

**Objective:**

This study examined the associations of self-compassion and self-efficacy with geriatric pain, postural awareness, health-promoting lifestyle behaviors, and quality of life in community-dwelling older adults in Turkey.

**Methods:**

In this cross-sectional study, 280 individuals were screened and 179 adults aged ≥65 years were included in the final analyses. Participants completed validated instruments including the Geriatric Pain Measure, Self-Compassion Scale, Self-Efficacy Scale, Postural Awareness Scale, Health-Promoting Lifestyle Profile (HPLP), and quality of life (WHOQOL-BREF).

**Results:**

Self-efficacy emerged as the most consistent predictor across outcomes. In the hierarchical model predicting quality of life, the final model explained 61.4% of the variance (R² = 0.614); self-efficacy (β = 0.340) was a positive predictor and geriatric pain (β=−0.346) a negative predictor, with health responsibility (β = 0.328) and interpersonal support (β = 0.181) also contributing (all p < 0.05). In the hierarchical models predicting HPLP subdimensions and the total score, the final models explained 21–40% of the variance (R² = 0.21–0.40); self-efficacy was a positive predictor across all subdimensions and the total score, self-compassion was the strongest predictor of nutrition (β = 0.436), and postural awareness was the strongest predictor of stress management (β = 0.300), whereas geriatric pain negatively predicted several domains. For geriatric pain, self-compassion (β=−0.205) and postural awareness (β=−0.212) were significant negative predictors, whereas self-efficacy was not (R² = 0.141). Bivariate associations were consistent with these models: HPLP total score showed the strongest correlation with quality of life (ρ = 0.575), which was also correlated with self-efficacy (ρ = 0.497) and self-compassion (ρ = 0.424); self-compassion was positively correlated with HPLP total score (ρ = 0.411) and self-efficacy (ρ = 0.376) and negatively correlated with geriatric pain (ρ=−0.267) (all p < 0.05).

**Conclusion:**

Self-efficacy was consistently associated with health-promoting behaviors and quality of life, while self-compassion and postural awareness showed more domain-specific associations with pain and lifestyle behaviors. These findings support a biopsychosocial assessment approach in older adults but do not establish that interventions targeting these resources would improve clinical outcomes.

## Introduction

Population ageing is accelerating globally, with the number of individuals aged 60 years and older projected to reach 2.1 billion by 2050 [1]. Turkey is experiencing a similarly rapid demographic transition: the proportion of adults aged 65 years and older increased to 9.9% in 2022 and is projected to rise to 25.6% by 2080 [2]. As populations age, it has become increasingly clear that health outcomes in later life are determined not only by biomedical factors but also by the interaction of biological capacity, psychological resources, and environmental and social conditions. This approach is conceptualized within the biopsychosocial model [3,4]. The WHO defines healthy ageing as the development and maintenance of functional ability that enables well-being in older age [5]. Accordingly, understanding how modifiable biopsychosocial resources contribute to functional capacity and quality of life has emerged as a central focus in contemporary geriatric research [6,7].

Chronic pain in later life is increasingly conceptualized as a multidimensional burden affecting physical functioning, behavioral engagement, and psychosocial well-being. With increasing life expectancy, chronic musculoskeletal pain conditions have become highly prevalent among older adults. It is estimated that 40–60% of older individuals experience chronic musculoskeletal pain [8]. In Turkey, 21.7% of community-dwelling adults aged 65 years and older report severe pain, which is frequently managed using passive strategies such as rest, massage, or heat application [9,10]. However, passive approaches often provide only temporary symptom relief and may not adequately address the behavioral and psychosocial determinants of long-term functional outcomes. In contrast, active biopsychosocial interventions—including structured exercise, movement-based rehabilitation, mind–body approaches, and self-management strategies—are widely recommended as first-line non-pharmacological treatments for chronic pain [11,12]. These interventions aim to improve long-term outcomes by simultaneously targeting physical function, behavioral engagement, and psychological adaptation.

Among psychological resources, self-compassion and self-efficacy have emerged as important self-regulatory constructs. Self-compassion, defined as a kind, accepting, and non-judgmental stance toward oneself in the face of difficulty [13], has been associated with reduced physiological stress responses, including modulation of the hypothalamic–pituitary–adrenal axis and lower daily cortisol levels [14]. Evidence also suggests that self-compassion-based interventions enhance psychological well-being in older adults [15]. Self-efficacy, defined as individuals’ belief in their ability to initiate and maintain behaviors [16], is a well-established determinant of health behavior change. It has also been associated with physiological regulation, including autonomic nervous system balance, heart rate variability, and modulation of the hypothalamic–pituitary–adrenal axis, suggesting a role in psychophysiological stress regulation [17,18]. In chronic musculoskeletal pain populations, higher self-efficacy is consistently associated with better physical functioning and lower disability [19]. Furthermore, health-related self-efficacy has been shown to mediate the association between self-compassion and pain-related disability [20]. Together, these findings suggest that self-compassion and self-efficacy may operate as modifiable psychological resources influencing both biological stress regulation and functional outcomes in later life.

Beyond these psychological resources, the way older adults perceive and regulate their own bodies may represent a further, more embodied form of self-regulation relevant to healthy aging. In this context, postural control and postural awareness represent critical domains for maintaining mobility and preventing falls in older adults. Postural control depends on the integration of somatosensory, vestibular, and visual inputs to maintain dynamic balance [21]. Postural awareness refers to the subjective capacity to monitor and regulate body posture during daily activities and may function as a behavioral and somatic self-regulation mechanism linking body perception with movement control and emotional regulation. Validation studies of the Postural Awareness Scale (PAS) have demonstrated associations between postural awareness, pain intensity, disability, and mental health indicators [22]. In addition, mind–body interventions have been shown to enhance postural awareness and reduce pain [23,24]. However, the extent to which psychological self-regulatory resources such as self-compassion and self-efficacy are associated with postural awareness in older adults remains largely unexplored. Examining postural awareness alongside these psychological resources may therefore clarify whether body-based and psychological forms of self-regulation are interrelated and how they relate to health behaviors and quality of life in later life.

Health-promoting behaviors, particularly regular physical activity, are fundamental determinants of functional independence and quality of life in older age. Systematic reviews consistently demonstrate that regular physical activity reduces the risk of functional decline, disability, and loss of independence in later life [25–27], and is positively associated with multiple domains of quality of life [28]. While self-efficacy is widely recognized as a central driver of sustained health-promoting behaviors, emerging meta-analytic evidence also indicates that self-compassion is positively associated with engagement in health-promoting behaviors, including physical activity and stress management [29].

In physiotherapy and geriatric rehabilitation, strengthening modifiable psychological resources has become increasingly relevant. Interventions targeting self-compassion and self-efficacy have demonstrated benefits in pain management, functional performance, and quality of life outcomes [19,20]. Conceptually, self-compassion and self-efficacy may serve as upstream biopsychosocial resources associated with both physical domains (such as pain intensity and postural awareness) and behavioral domains (health-promoting behaviors), ultimately shaping overall health-related quality of life [30–32]. However, these variables are typically examined in isolation in the literature. Studies simultaneously investigating psychological self-regulatory resources, postural awareness, health behaviors, and quality of life within a unified biopsychosocial framework remain limited, particularly among community-dwelling older adults.

To our knowledge, no study has comprehensively investigated the associations between self-compassion, self-efficacy, pain intensity, postural awareness, health-promoting behaviors, and quality of life within a unified biopsychosocial model among older adults in Turkey. Understanding these interrelationships is essential for designing multidimensional geriatric rehabilitation strategies that extend beyond symptom management and address modifiable psychological and behavioral pathways [33]. Therefore, the present study aimed to examine these associations in Turkish older adults. Accordingly, we tested the following hypotheses:

(1) Higher self-compassion and self-efficacy would be associated with lower geriatric pain.
(2) Higher self-compassion and self-efficacy would be associated with greater postural awareness.
(3) Higher self-compassion and self-efficacy would be associated with stronger engagement in health-promoting behaviors.
(4) Higher self-compassion and self-efficacy would be associated with better quality of life.
(5) Self-compassion, self-efficacy, and postural awareness would be significant predictors of these outcomes in multivariable models.

## Methods

### Study design

A cross-sectional and correlational design was used in this study. Correlation analyses were conducted to examine the relationships between the study variables, while multiple and hierarchical multiple regression analyses were used to identify significant predictive factors. A convenience sampling strategy was employed in the study. Participants were selected from among individuals who attended Acıbadem Healthcare Group for their routine annual health check-ups during the data collection period. Data were collected between 15 January 2026 and 1 March 2026 in a supervised clinical setting, using self-administered paper-based questionnaires completed by participants during face-to-face sessions. Prior to participation, all individuals were informed about the study both verbally and in writing, and written informed consent was obtained from those who agreed to take part.

The study protocol was approved by the Acıbadem University and Acıbadem Healthcare Institutions Medical Research Ethics Committee (ATADEK: 2025-11/428). All procedures were conducted in accordance with the Declaration of Helsinki. The study was prospectively registered at ClinicalTrials.gov (Registration No: NCT07370090) on 15 January 2026, and participant enrolment commenced thereafter, continuing until 1 March 2026.

### Participants

Community-dwelling adults aged 65 years and older attending routine annual health check-ups at several hospitals within the Acıbadem Healthcare Group network during the data collection period were invited to participate. Additionally, individuals aged 65 and older accompanying patients as companions or visitors were also invited. Eligible participants who met inclusion criteria and provided informed consent were enrolled.

**Fig 1:**
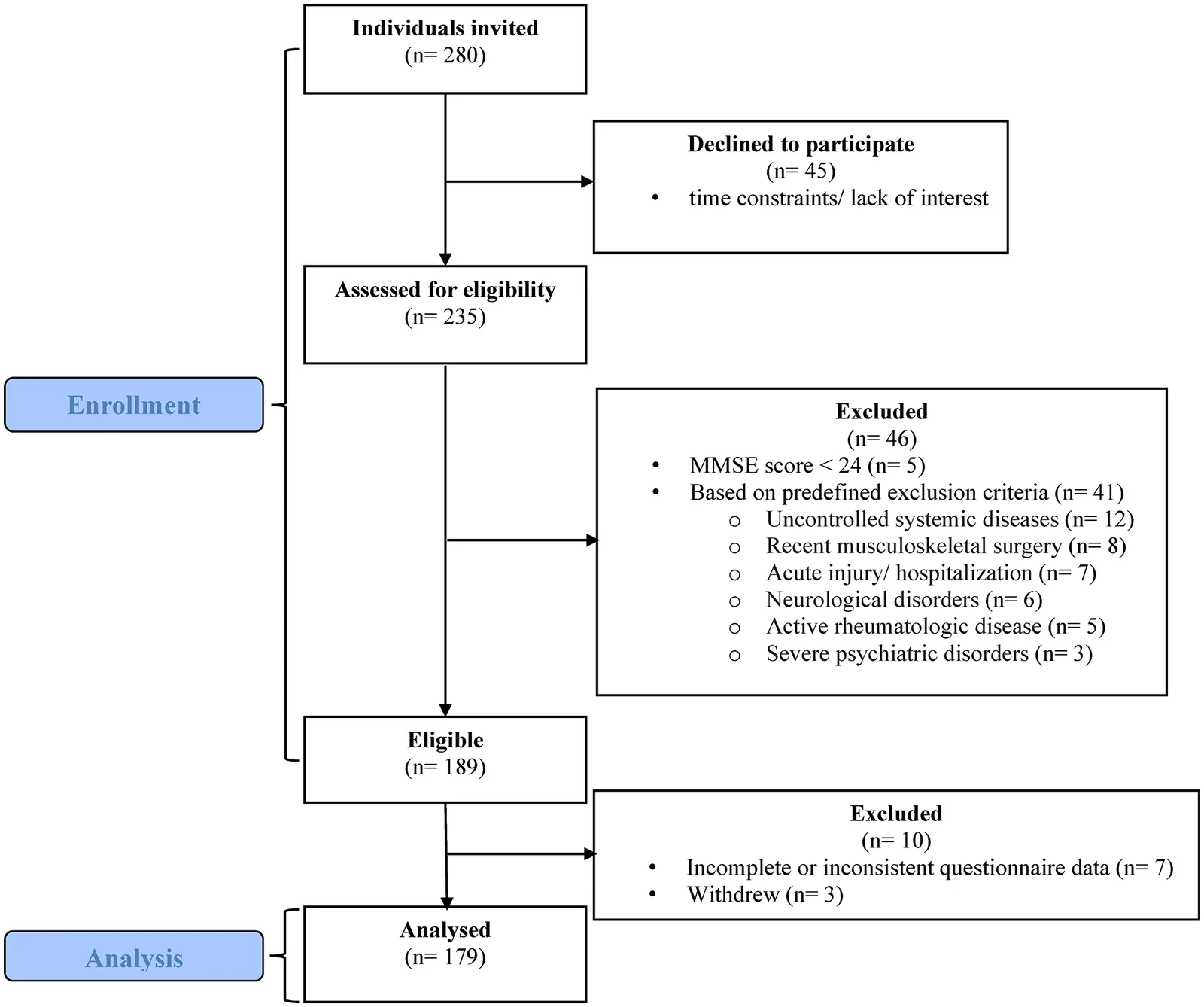
Flow diagram of participant recruitment, eligibility assessment, exclusions, and inclusion in the final analysis.

Sample size estimation was performed a priori using G*Power 3.1.9.7 (F tests; Linear multiple regression: Fixed model, R² deviation from zero). Based on Kim and Ko’s [34] reported effect size examining self-compassion and psychological outcomes in older adults, a small-to-moderate effect size (Cohen’s f² = 0.10) was assumed. With an alpha of 0.05, power of 0.80, and eight independent variables, the minimum sample size was 159. To accommodate potential incomplete or ineligible data, the target sample was increased by approximately 10% to 175 participants. The achieved sample size (n = 179) exceeded the a priori estimate. A sensitivity analysis conducted in G*Power (linear multiple regression: fixed model, R² deviation from zero; α = 0.05, power = 0.80, eight predictors) indicated that the study was able to detect effect sizes as small as f² = 0.088, corresponding to a small effect, supporting adequate statistical power for the primary regression models. A flow diagram of participant recruitment and inclusion is presented in Fig 1, following STROBE recommendations.

Inclusion criteria were as follows:

- Age ≥ 65 years
- Mini-Mental State Examination score ≥ 24
- Living independently in the community

Exclusion criteria were as follows:

- Diagnosed severe cognitive impairment (e.g., dementia, Alzheimer’s disease)
- Severe psychiatric disorders
- Uncontrolled major systemic chronic diseases
- Major musculoskeletal surgery within the previous 6 months
- Acute musculoskeletal injury or hospitalization within the previous 3 months
- Neurological disorders affecting mobility or balance
- Active inflammatory rheumatologic disease
- Incomplete or inconsistent questionnaire responses

Participants with well-controlled chronic conditions (e.g., hypertension, hyperlipidemia, type 2 diabetes without functional limitation) were included.

## Measurements

### The study variables were assessed using standardized and validated instruments

#### Geriatric pain measure.

Pain was assessed using the Geriatric Pain Measure (GPM-24), a multidimensional instrument developed by Ferrell et al. [35] to evaluate pain in older adults. The scale comprises 24 items: 22 dichotomous (yes/no) items assessing pain presence and impact across various daily activities, and 2 items measuring pain intensity on an 11-point numerical rating scale (0–10). The total score is calculated by summing the relevant item responses and converting the raw score to a 0–100 scale by multiplying by 2.38. Higher scores indicate greater pain severity (<30 mild; 30–69 moderate; ≥ 70 severe). However, all statistical analyses were conducted using continuous scores. Subscale scores (pain intensity, ambulation-related pain, strenuous activity-related pain, other activity-related pain, and pain disengagement) were calculated separately and analyzed as continuous variables. The Turkish version’s validity and reliability were established by Dursun and Bektaş [36], with construct validity supported by factor loadings ranging from 0.40 to 0.87 and good internal consistency (Cronbach’s α = 0.85) for the total scale; subscale Cronbach’s alpha coefficients ranged from 0.67 to 0.93.

#### Self-compassion scale.

Self-compassion was assessed using the Self-Compassion Scale (SCS) developed by Neff [13]. The scale consists of 26 items rated on a 5-point Likert scale ranging from 1 (almost never) to 5 (almost always). The scale includes six subscales: Self-kindness, self-judgment, common humanity, isolation, mindfulness, and over-identification. Negative subscales (self-judgment, isolation, and over-identification) are reverse-scored. Subscale means are computed, and the total self-compassion score is obtained by averaging these six subscale scores. Higher scores indicate greater self-compassion, with scores between 1–2.5 indicating low, 2.5–3.5 moderate, and 3.5–5 high levels of self-compassion. The Turkish adaptation by Akin et al. [37] demonstrated internal consistency coefficients ranging from α = .72 to .80, test–retest reliability between .56 and .69, and corrected item–total correlations from .48 to .71, supporting the scale’s reliability and construct validity.

#### Self-efficacy scale.

Self-efficacy was assessed using the Self-Efficacy Scale developed by Sherer et al. [38]. The scale consists of 23 items rated on a 5-point Likert scale assessing individuals’ perceived competence in performing behaviors. It comprises four subdimensions: initiation of behavior, maintenance of behavior, completion of behavior, and coping with obstacles. Fourteen items are reverse-scored. Higher total scores indicate greater perceived self-efficacy. The Turkish adaptation by Gözüm and Aksayan [39] reported satisfactory psychometric properties, including internal consistency (Cronbach’s α = 0.81) and test–retest reliability (r = 0.92).

#### Postural awareness scale.

Postural awareness was assessed using the Postural Awareness Scale (PAS) [22], a self-report instrument measuring individuals’ awareness of their body posture during daily activities. The original scale comprises 12 items rated on a 7-point Likert scale ranging from 1 (“not at all true for me”) to 7 (“completely true for me”). The Turkish validity and reliability study by Dursun and Önen [40] resulted in the removal of the 12th item due to low factor loading in confirmatory factor analysis, yielding an 11-item, two-factor structure. The Turkish version demonstrated satisfactory internal consistency (total α = .854; factor 1 α = .886; factor 2 α = .777) and good test–retest reliability over a two-week interval (r = .831). Higher total scores indicate greater postural awareness. The present study used the total PAS score from the 11-item Turkish version for statistical analyses.

#### Healthy lifestyle behaviors.

Healthy lifestyle behaviors were assessed using the Health-Promoting Lifestyle Profile (HPLP), developed by Walker et al. [41]. The scale measures the frequency of engagement in health-promoting behaviors and has been widely applied across diverse populations. The Turkish adaptation and validation was performed by Esin [42]. The Turkish version comprises 48 positively worded items rated on a 4-point Likert scale ranging from 1 (“never”) to 4 (“routinely”). It includes six subdimensions: self-actualization, health responsibility, exercise, nutrition, interpersonal support, and stress management. No reverse scoring is necessary. Total scores range from 48 to 192, with higher scores indicating more frequent engagement in health-promoting behaviors. The Turkish validation study reported excellent overall test–retest reliability (r = 0.99) and subscale reliability coefficients between 0.97 and 0.99. Item–total correlations ranged from 0.27 to 0.55, supporting the scale’s reliability.

#### Health-related quality of life.

Health-related quality of life was assessed using the World Health Organization Quality of Life – Brief Version (WHOQOL-BREF), a 26-item self-report instrument developed by the WHO WHOQOL Group [43]. The WHOQOL-BREF evaluates quality of life across four domains: physical health, psychological health, social relationships, and environmental health. Each item is rated on a 5-point Likert scale. Domain scores are transformed to a 0–100 scale, with higher scores indicating better quality of life. The Turkish validity and reliability study was conducted by Eser et al. [44], with the psychometric properties of the Turkish version found to be satisfactory.

### Statistical analysis

All statistical analyses were performed using jamovi (Version 2.3, The Jamovi Project, 2023). Statistical significance was set at p < 0.05 (two-tailed). Continuous variables were expressed as means ± standard deviations; categorical variables as frequencies and percentages. Normality was assessed using the Shapiro–Wilk test and visual inspection of Q–Q plots. Due to non-normal distributions, group comparisons were conducted using the Mann–Whitney U test (gender) and the Kruskal–Wallis H test with Dunn’s post hoc comparisons (chronic disease groups). To address the possibility of Type I error inflation arising from multiple gender comparisons, a Bonferroni-corrected sensitivity analysis was additionally applied. Corrected thresholds were calculated separately for each measure as 0.05 divided by the number of comparisons within that scale (total score and subdimensions); comparisons surviving correction are indicated in Table 2.

Bivariate associations among study variables were examined using Spearman’s rank correlation coefficients. Correlation strength was interpreted as follows: 0.00–0.19 very weak, 0.20–0.39 weak, 0.40–0.59 moderate, 0.60–0.79 strong, and ≥0.80 very strong [45].

Multiple linear regression analyses examined the associations of self-compassion and self-efficacy with postural awareness, and the associations of self-compassion, self-efficacy, and postural awareness with geriatric pain. Multicollinearity was assessed using variance inflation factors (VIF < 5). Although several raw variables deviated from normality on the Shapiro–Wilk test, multiple linear regression is robust to non-normality of raw predictors and outcomes, as the relevant assumption concerns the model residuals rather than the raw distributions. Regression assumptions were therefore verified through residual diagnostics: normality of residuals was assessed using Q–Q plots, homoscedasticity through residual-versus-fitted plots, and independence of errors using the Durbin–Watson statistic (1.94 and 1.96 in the geriatric pain and quality of life models, respectively, both within the conventionally acceptable range of 1.5–2.5). These diagnostics indicated that the residuals were approximately normally distributed, that the assumption of constant variance was adequately met, and that error independence was not violated. Hierarchical multiple regression analyses were conducted to identify predictors of healthy lifestyle behaviors and quality of life. The order of variable entry followed the conceptual logic of the biopsychosocial model, moving from non-modifiable background characteristics to clinical burden and finally to modifiable psychological and body-awareness resources, so that the incremental contribution of each block could be evaluated over and above the preceding ones. For healthy lifestyle behaviors, demographic variables (background characteristics) were entered in Step 1, geriatric pain (clinical burden) in Step 2, and psychological–body-awareness resources (self-compassion, self-efficacy, postural awareness) in Step 3. For quality of life, demographic variables and geriatric pain were entered in Step 1 as background and clinical factors; health-promoting lifestyle subdimensions and postural awareness were entered in Step 2 as behavioral and body-awareness factors; and self-efficacy and self-compassion were entered in Step 3 to test whether psychological resources explained additional variance beyond behavioral and clinical factors. Changes in R² and F-change statistics were evaluated at each step. The adequacy of the predictor-to-observation ratio was considered in model specification. In the most complex model (quality of life, Step 3), 13 predictors were estimated with 179 observations, yielding approximately 13.8 cases per predictor, which exceeds the commonly recommended minimum of 10 cases per predictor for stable regression estimates [46]. Effect sizes for explained variance were interpreted according to Cohen’s guidelines (R² = 0.02 small, 0.13 moderate, 0.26 large) [47]. Internal consistency of all scales was assessed using Cronbach’s alpha (α ≥ 0.70 considered acceptable) [48]. To examine potential common-method variance, given that all constructs were assessed via self-report at a single time point, Harman’s single-factor test was conducted by entering the subscale scores of all study measures into an exploratory factor analysis constrained to a single unrotated factor [49].

Given the exploratory, hypothesis-generating nature of this study and the number of bivariate correlations and regression models examined, the possibility of Type I error inflation is acknowledged. To balance Type I and Type II error risks, the primary correlation and regression analyses are reported without formal correction for multiple comparisons, while a Bonferroni-corrected sensitivity analysis is provided for the gender comparisons. Statistically significant associations that are modest in magnitude should therefore be interpreted with appropriate caution.

## Results

### Participant characteristics

As illustrated in Fig 1, a total of 179 community-dwelling older adults were included in the final analyses after the recruitment and eligibility screening process. The mean age was 72.51 ± 5.96 years (range: 65–98), with 57% women. Most participants were married (81%) and retired (50.8%). Approximately 30.2% had no formal education, whereas 21.2% were university graduates. Regular medication use was reported by 77.7%, and 64.8% had at least one chronic disease. Detailed demographic characteristics are presented in Table 1.

**Table 1:** Characteristics of participants.

Demographic Information		n	%
**Gender**	Female	102	57.0
	Male	77	43.0
**Marital Status**	Married	145	81.0
	Single	34	19.0
**Occupation**	Retired	91	50.8
	Employed	33	18.4
	Housewife	55	30.7
**Education Level**	No education	54	30.2
	Primary school	26	14.5
	Middle school	14	7.8
	High school	47	26.3
	University	38	21.2
**Smoking Status**	Still smoking	40	22.3
	Never smoked	73	40.8
	Quit smoking	66	36.9
**Medication Use**	Yes	139	77.7
	No	40	22.3
**Presence of Disease**	None	63	35.2
	HT	35	19.6
	T2DM	21	11.7
	Other	60	33.5

HT = Hypertension; T2DM = Type 2 Diabetes Mellitus

### Descriptive statistics of main study variables

Descriptive statistics are summarized in Table 2. Self-compassion levels were moderate (3.25 ± 0.66), with relatively higher subscale scores for self-judgment (3.55 ± 0.78) and isolation (3.64 ± 0.91). Self-efficacy scores (82.50 ± 13.08) indicated adequate perceived competence, with higher scores in behavioral initiation (29.30 ± 6.39) and maintenance (25.67 ± 4.85). Postural awareness was 33.51 ± 8.93, with a higher mean score for the attention subscale (21.36 ± 8.47) than for the ease subscale (12.15 ± 3.62). Geriatric Pain Measure scores (39.32 ± 21.11) reflected moderate pain levels. Subscales for pain intensity (7.37 ± 4.31) and pain disengagement (20.65 ± 11.48) were elevated. Healthy lifestyle behaviors (129.54 ± 21.40) indicated moderate to good engagement, with higher scores in self-actualization (34.66 ± 6.23) and interpersonal support (19.86 ± 3.93), and lower scores in exercise (12.94 ± 2.78). Quality of life (67.32 ± 15.20) was moderate, highest in the environmental domain (72.17 ± 17.80) and lowest in social relationships (61.31 ± 18.71). Physical health (68.79 ± 19.56) and psychological domain (67.02 ± 18.64) scores were similar.

**Table 2:** Means of variables and comparison by gender.

Measurement	Mean ± SD	Min–Max	Male (n = 77) Mean ± SD	Female (n = 102) Mean ± SD	Mann-Whitney U	p	Rank Biserial
**Self-Compassion Scale (Total Score)**	3.25 ± 0.66	1.16–4.73	3.52 ± 0.62	3.04 ± 0.62	2168	<0.001***ª	−0.448
Self-kindness	2.98 ± 0.97	1.00–5.00	3.21 ± 1.08	2.81 ± 0.84	2891	0.002**ª	−0.264
Self-judgment	3.55 ± 0.78	1.20–5.00	3.86 ± 0.68	3.31 ± 0.76	2189	<0.001***	−0.443
Common humanity	3.02 ± 0.83	1.25–5.00	3.07 ± 0.90	2.98 ± 0.78	3642	0.404	−0.073
Isolation	3.64 ± 0.91	1.00–5.00	3.85 ± 0.79	3.48 ± 0.97	2990	0.006**ª	−0.239
Mindfulness	3.10 ± 1.01	1.00–5.00	3.45 ± 1.14	2.84 ± 0.81	2364	<0.001***ª	−0.398
Over-identification	3.19 ± 0.97	1.00–5.00	3.66 ± 0.79	2.83 ± 0.94	1975	<0.001***ª	−0.497
**Self-Efficacy Scale (Total Score)**	82.50 ± 13.08	46–110	85.05 ± 13.22	80.58 ± 12.70	3351	0.093	−0.147
Initiation of behavior	29.30 ± 6.39	14–40	30.2 ± 7.04	28.6 ± 5.79	3211	0.037*	−0.182
Maintenance of behavior	25.67 ± 4.85	12–35	25.7 ± 5.62	25.6 ± 4.21	3812	0.737	−0.029
Completion of behavior	18.74 ± 4.42	9–25	19.7 ± 4.44	18.0 ± 4.30	3073	0.013*	−0.218
Coping with obstacles	8.79 ± 2.58	4–15	9.48 ± 2.72	8.27 ± 2.35	2941	0.004**ª	−0.251
**Postural Awareness Scale (Total Score)**	33.51 ± 8.93	11–50	32.32 ± 9.02	34.40 ± 8.81	3311	0.072	0.157
Ease	12.15 ± 3.62	5–20	11.4 ± 3.05	12.7 ± 3.92	3007	0.007**ª	0.234
Attention	21.36 ± 8.47	6–42	20.9 ± 9.49	21.7 ± 7.64	3612	0.359	0.080
**Geriatric Pain Measure (Total Score)**	39.32 ± 21.11	0–83.30	34.06 ± 18.74	43.28 ± 22.00	2976	0.006**ª	0.242
Pain intensity	7.37 ± 4.31	0–14.28	6.55 ± 4.09	7.98 ± 4.39	3196	0.031*	0.186
Pain disengagement	20.65 ± 11.48	0–45.22	18.8 ± 11.0	22.0 ± 11.7	3352	0.093	0.146
Ambulation-related pain	5.23 ± 3.78	0–9.52	4.88 ± 3.74	5.48 ± 3.80	3565	0.277	0.092
Strenuous activity-related pain	2.14 ± 2.08	0–7.14	1.27 ± 1.47	2.80 ± 2.23	2416	<0.001***ª	0.385
Other activity-related pain	3.94 ± 3.53	0–11.90	2.53 ± 2.87	4.99 ± 3.62	2381	<0.001***ª	0.394
**HPLP (Total Score)**	129.54 ± 21.40	79–186	130.84 ± 22.28	128.56 ± 20.77	3584	0.318	−0.087
Self-actualization	34.66 ± 6.23	19–52	35.68 ± 6.57	33.89 ± 5.88	3216	0.038*	−0.181
Health responsibility	25.12 ± 5.31	15–38	25.49 ± 5.06	24.83 ± 5.50	3562	0.287	−0.093
Exercise	12.94 ± 2.78	6–20	12.82 ± 2.66	13.03 ± 2.87	3842	0.803	0.022
Nutrition	17.42 ± 2.93	6–24	17.30 ± 2.80	17.51 ± 3.03	3781	0.668	0.037
Interpersonal support	19.86 ± 3.93	10–28	20.10 ± 4.11	19.68 ± 3.81	3640	0.402	−0.073
Stress management	19.77 ± 3.20	12–28	19.71 ± 3.51	19.81 ± 2.97	3666	0.445	0.067
**WHOQOL-BREF(Total Score)**	67.32 ± 15.20	28.76–98.21	70.77 ± 14.53	64.72 ± 15.24	3001	0.007**ª	−0.236
Physical health	68.79 ± 19.56	17.86–100	72.26 ± 18.15	66.18 ± 20.26	3285	0.061	−0.164
Psychological domain	67.02 ± 18.64	0–100	71.43 ± 17.70	63.68 ± 18.72	3078	0.013*	−0.216
Social relationships	61.31 ± 18.71	8.33–100	62.01 ± 16.91	60.78 ± 20.02	3667	0.445	−0.066
Environmental domain	72.17 ± 17.80	28.13–100	77.39 ± 17.66	68.23 ± 16.95	2617	<0.001***ª	−0.334

SD = Standard deviation; HPLP = Health-Promoting Lifestyle Profile; WHOQOL-BREF = World Health Organization Quality of Life – BREF; * p < 0.05, ** p < 0.01, *** p < 0.001

Superscript “a” denotes comparisons that remained significant after Bonferroni correction (corrected threshold = 0.05 / number of comparisons per scale).

Harman’s single-factor test indicated that a single factor accounted for 30.2% of the total variance, well below the 50% threshold, suggesting that common-method variance was unlikely to substantially bias the observed associations.

In the present sample, internal consistency was acceptable to excellent across all instruments (Cronbach’s α: Self-Compassion Scale = .91, Self-Efficacy Scale = .83, Geriatric Pain Measure = .84, Health-Promoting Lifestyle Profile = .93, Postural Awareness Scale = .74, WHOQOL-BREF = .93).

### Differences by gender

Gender differences were analyzed using the Mann–Whitney U test (Table 2). Men had significantly higher self-compassion scores (3.52 ± 0.62) than women (3.04 ± 0.62) (U = 2168, p < 0.001), with significant differences also observed in the self-kindness (U = 2891, p = 0.002), isolation (U = 2990, p = 0.006), mindfulness (U = 2364, p < 0.001), and over-identification (U = 1975, p < 0.001) subscales. Women reported significantly higher geriatric pain scores (43.28 ± 22.00) than men (34.06 ± 18.74) (U = 2976, p = 0.006). No significant gender differences were found in total self-efficacy scores (U = 3351, p = 0.093); however, men scored significantly higher on the initiation of behavior (U = 3211, p = 0.037), completion of behavior (U = 3073, p = 0.013), and coping with obstacles (U = 2941, p = 0.004) subscales. No significant gender differences were found in total healthy lifestyle behaviors (U = 3584, p = 0.318); however, men scored higher on the self-actualization subscale (35.68 ± 6.57 vs. 33.89 ± 5.88) (U = 3216, p = 0.038). Other healthy lifestyle subdimensions did not differ significantly by gender (p > 0.05). Postural awareness total scores did not differ significantly by gender (U = 3311, p = 0.072); however, a significant gender difference was observed in the ease subdimension, with women scoring higher than men (12.73 ± 3.92 vs. 11.38 ± 3.05; U = 3007, p = 0.007). Quality of life total scores were higher in men (70.77 ± 14.53) than women (64.72 ± 15.24) (U = 3001, p = 0.007). Domain-level analyses revealed significantly higher psychological (U = 3078, p = 0.013) and environmental (U = 2617, p < 0.001) scores in men, with no significant differences in physical health (U = 3285, p = 0.061) or social relationships (U = 3667, p = 0.445).

After Bonferroni correction for multiple gender comparisons, the principal differences remained statistically significant, including total self-compassion and its self-kindness, self-judgment, isolation, mindfulness, and over-identification subscales; total geriatric pain together with strenuous activity–related and other activity–related pain; the coping with obstacles subscale of self-efficacy; the ease subscale of postural awareness; and total quality of life with its environmental domain. In contrast, several differences that were significant at the uncorrected level did not survive correction, namely the initiation of behavior and completion of behavior subscales of self-efficacy, pain intensity, the self-actualization subscale of health-promoting behaviors, and the psychological domain of quality of life. These results should therefore be interpreted with greater caution.

### Correlation analysis among study variables

Spearman’s rank correlations among the primary study variables are presented in Table 3, while the complete correlation matrix including the HPLP and WHOQOL-BREF subdimensions is provided in S1 Table. Self-compassion was positively correlated with self-efficacy (ρ = .376, p < 0.001), healthy lifestyle behaviors (ρ = .411, p < 0.001), postural awareness (ρ = .215, p = 0.004), and quality of life (ρ = .424, p < 0.001), and negatively correlated with geriatric pain (ρ = −.267, p < 0.001). Self-efficacy was positively correlated with healthy lifestyle behaviors (ρ = .348, p < 0.001) and quality of life (ρ = .497, p < 0.001), and weakly negatively correlated with geriatric pain (ρ = −.159, p = 0.033) and age (ρ = −.165, p = 0.027).

**Table 3:** Spearman’s rank correlations among the primary study variables.

Variable	Statistic	Age	Self-Compassion Scale	Self-Efficacy Scale	Postural Awareness Scale	Geriatric Pain Measure	HPLP total score	WHOQOL-BREF total score
**Age**	p	—						
	ρ	—						
**Self-Compassion Scale**	p	0.405	—					
	ρ	−0.063	—					
**Self-Efficacy Scale**	p	0.027	<0.001	—				
	ρ	−0.165*	0.376***	—				
**Postural Awareness Scale**	p	0.092	0.004	0.586	—			
	ρ	−0.126	0.215**	0.041	—			
**Geriatric Pain Measure**	p	0.898	<0.001	0.033	0.001	—		
	ρ	0.010	−0.267***	−0.159*	−0.238**	—		
**HPLP total score**	p	0.568	<0.001	<0.001	<0.001	<0.001	—	
	ρ	−0.043	0.411***	0.348***	0.272***	−0.351***	—	
**WHOQOL-BREF total score**	p	0.790	<0.001	<0.001	0.002	<0.001	<0.001	—
	ρ	−0.020	0.424***	0.497***	0.227**	−0.529***	0.575***	—

ρ = Spearman’s rank correlation coefficient; HPLP = Health-Promoting Lifestyle Profile; WHOQOL-BREF = World Health Organization Quality of Life–Brief Version. *p < .05, **p < .01, ***p < .001.

Geriatric pain was negatively correlated with all key psychosocial, lifestyle, and quality of life variables (all p < 0.05). Postural awareness was positively associated with healthy lifestyle behaviors (ρ = .272, p < 0.001) and quality of life (ρ = .227, p = 0.002), and negatively with geriatric pain (ρ = −.238, p = 0.001). Healthy lifestyle behaviors showed the strongest positive correlation with quality of life (ρ = .575, p < 0.001). The HPLP subdimensions were moderately to strongly intercorrelated (ρ = .544–.851) and were strongly correlated with the total HPLP score (ρ = .758–.943; all p < .001; S1 Table).

### Multiple linear regression analyses

Multiple linear regression models examined the predictive roles of self-compassion, self-efficacy, and postural awareness (Table 4).

**Table 4:** Multiple linear regression analysis results for predictors of geriatric pain measure and postural awareness scale.

Geriatric Pain Measure							
	B	SE	β	t	p	95% CI	VIF
Intercept	94.88	11.41		8.32	<0.001	[72.36, 117.40]	
Self-Compassion Scale	−6.52	2.44	−0.205	−2.67	0.008	[-11.34, -1.70]	1.20
Self-Efficacy Scale	−0.21	0.12	−0.132	−1.76	0.080	[-0.45, 0.03]	1.15
Postural Awareness Scale	−0.16	0.05	−0.212	−2.91	0.004	[-0.27, -0.05]	1.05
**Model Fit**	R = 0.375	R² = 0.141	Adj. R² = 0.126	F(3, 175) = 9.55	p < 0.001		
**Postural Awareness Scale**							
	B	SE	β	t	p	95% CI	VIF
Intercept	28.09	4.60		6.10	<0.001	[19.01, 37.18]	
Self-Compassion Scale	3.16	1.06	0.234	2.99	0.003	[1.07, 5.25]	1.14
Self-Efficacy Scale	−0.06	0.05	−0.086	−1.09	0.275	[-0.16, 0.05]	1.14
**Model Fit**	R = 0.220	R² = 0.048	Adj. R² = 0.037	F(2, 176) = 4.46	p = 0.013		

B = Unstandardized regression coefficient; SE = Standard error; β = Standardized regression coefficient; CI = Confidence interval; VIF = Variance inflation factor.

For geriatric pain, the model was significant (F(3,175) = 9.55, p < 0.001), explaining 14.1% of the variance (R² = 0.141, adjusted R² = 0.126). Variance inflation factor values ranged from 1.05 to 1.20, indicating no multicollinearity. Self-compassion (β = −0.205, p = 0.008) and postural awareness (β = −0.212, p = 0.004) were significant negative predictors of geriatric pain, while self-efficacy was not (β = −0.132, p = 0.080).

For postural awareness, the model was significant (F(2,176) = 4.46, p = 0.013), explaining 4.8% of variance (R² = 0.048, adjusted R² = 0.037). VIF = 1.14 indicated no multicollinearity, and diagnostic plots supported model assumptions. Self-compassion was a significant positive predictor (β = 0.234, p = 0.003), whereas self-efficacy was not significant (β = −0.086, p = 0.275).

### Hierarchical multiple regression analysis predicting healthy lifestyle behaviors

Separate hierarchical multiple regression analyses were conducted for each HPLP subdimension and for the total HPLP score (Table 5). Demographic variables were entered in Step 1, geriatric pain in Step 2, and psychological variables (self-compassion, self-efficacy, and postural awareness) in Step 3. Only significant predictors (p < .05) are presented in the table.

**Table 5:** Hierarchical regression models for healthy lifestyle behavior subdimensions and total score.

HPLP Subdimension	Model 1 R²	Model 2 R² (ΔR²)	Model 3 R² (ΔR²)	Significant Predictor	B	95% CI for B	β	p
Self-Actualization	0.028	0.115 (0.087***)	0.256 (0.141***)	Self-Compassion	2.262	[0.81, 3.71]	0.240	0.002**
				Self-Efficacy	0.091	[0.02, 0.16]	0.190	0.009**
				Postural Awareness	0.119	[0.02, 0.22]	0.170	0.018*
				Geriatric Pain	−0.050	[−0.09, −0.01]	−0.168	0.023*
Health Responsibility	0.019	0.110 (0.091***)	0.244 (0.134***)	Geriatric Pain	−0.054	[−0.09, −0.02]	−0.214	0.004**
				Self-Efficacy	0.083	[0.02, 0.14]	0.203	0.006**
				Postural Awareness	0.112	[0.03, 0.20]	0.188	0.010*
				Self-Compassion	1.350	[0.10, 2.59]	0.168	0.034*
Exercise	0.007	0.152 (0.145***)	0.247 (0.095***)	Geriatric Pain	−0.037	[−0.06, −0.02]	−0.277	<0.001***
				Self-Compassion	0.832	[0.18, 1.48]	0.198	0.012*
				Gender	0.987	[0.17, 1.80]	0.176	0.018*
				Postural Awareness	0.049	[0.01, 0.09]	0.159	0.028*
				Self-Efficacy	0.031	[0.00, 0.06]ᵃ	0.145	0.047*
Nutrition	0.003	0.175 (0.172***)	0.399 (0.224***)	Self-Compassion	1.928	[1.32, 2.54]	0.436	<0.001***
				Geriatric Pain	−0.041	[−0.06, −0.02]	−0.296	<0.001***
				Gender	1.702	[0.93, 2.47]	0.289	<0.001***
				Self-Efficacy	0.040	[0.01, 0.07]	0.179	0.006**
Stress Management	0.003	0.059 (0.056**)	0.214 (0.155***)	Postural Awareness	0.108	[0.06, 0.16]	0.300	<0.001***
				Self-Efficacy	0.051	[0.01, 0.09]	0.208	0.006**
Interpersonal Support	0.016	0.051 (0.035*)	0.230 (0.179***)	Self-Efficacy	0.102	[0.06, 0.14]	0.338	<0.001***
				Self-Compassion	1.317	[0.39, 2.24]	0.222	0.005**
HPLP Total Score	0.009	0.122 (0.113***)	0.296 (0.174***)	Self-Efficacy	0.399	[0.17, 0.63]	0.244	<0.001***
				Self-Compassion	7.872	[3.03, 12.72]	0.244	0.002**
				Geriatric Pain	−0.201	[−0.34, −0.06]	−0.198	0.006**
				Postural Awareness	0.433	[0.10, 0.76]	0.181	0.010*

Model 1 = Demographic variables (Gender, Age, Chronic disease); Model 2 = Model 1 + Geriatric Pain; Model 3 = Model 2 + Psychosocial variables (Self-Compassion, Self-Efficacy, Postural Awareness). B = Unstandardized regression coefficient; β = Standardized regression coefficient; p = significance level. HPLP = Health-Promoting Lifestyle Profile. Gender coded as 0 = male, 1 = female. Only significant predictors (p < 0.05) are presented. *p < 0.05, **p < 0.01, ***p < 0.001.

Step 1 explained minimal variance (R² = 0.003–0.028). The addition of geriatric pain in Step 2 significantly increased explained variance across outcomes, particularly for nutrition (ΔR² = 0.172) and exercise (ΔR² = 0.145). In the final models (R² range = 0.214–0.399), self-efficacy was a significant positive predictor for all subdimensions and the total HPLP score. Self-compassion was positively associated with self-actualization, health responsibility, exercise, nutrition (strongest effect; β = 0.436), interpersonal support, and the total score. Geriatric pain was a significant negative predictor of self-actualization, health responsibility, exercise, nutrition, and the total score. Postural awareness positively predicted self-actualization, health responsibility, exercise, stress management (β = 0.300), and the total score. Gender showed domain-specific effects, predicting exercise and nutrition.

### Hierarchical multiple regression analysis predicting quality of life

A three-step hierarchical regression analysis was conducted to identify biopsychosocial predictors of health-related quality of life (Table 6).

**Table 6:** Hierarchical regression models predicting health-related quality of life.

Predictor	B	95% CI for B	SE	β	t	p
**Model 1 (R² = 0.291, Adj. R² = 0.275, F(4,174)=17.9, p < .001)**						
Gender	−2.802	[−6.78, 1.18]	2.016	−0.092	−1.390	0.166
Age	0.024	[−0.30, 0.35]	0.163	0.009	0.144	0.886
Chronic disease	−0.706	[−2.23, 0.81]	0.770	−0.059	−0.917	0.361
Geriatric pain measure	−0.362	[−0.46, −0.27]	0.048	−0.502	−7.599	<.001***
**Model 2 (R² = 0.519, Adj. R² = 0.488, ΔR² = 0.228, Fchange(7,167)=11.3, p < .001)**						
Gender	−3.219	[−6.82, 0.38]	1.823	−0.105	−1.765	0.079
Age	0.066	[−0.21, 0.34]	0.141	0.026	0.468	0.640
Chronic disease	−0.505	[−1.80, 0.79]	0.654	−0.042	−0.772	0.441
Geriatric pain measure	−0.270	[−0.36, −0.18]	0.046	−0.375	−5.817	<.001***
Postural awareness	0.082	[−0.12, 0.29]	0.104	0.048	0.788	0.432
HPLP – Self-actualization	−0.389	[−1.02, 0.24]	0.321	−0.160	−1.214	0.226
HPLP – Health responsibility	0.931	[0.30, 1.56]	0.319	0.325	2.922	0.004**
HPLP – Exercise	−0.755	[−1.79, 0.28]	0.525	−0.138	−1.438	0.152
HPLP – Nutrition	0.167	[−0.75, 1.09]	0.466	0.032	0.358	0.721
HPLP – Stress management	0.551	[−0.28, 1.38]	0.421	0.116	1.309	0.192
HPLP – Interpersonal support	1.273	[0.61, 1.94]	0.338	0.329	3.772	<.001***
**Model 3 (R² = 0.614, Adj. R² = 0.584, ΔR² = 0.095, Fchange(2,165)=20.3, p < .001)**						
Gender	−0.901	[−4.48, 2.68]	1.811	−0.029	−0.497	0.620
Age	0.159	[−0.09, 0.41]	0.129	0.063	1.239	0.217
Chronic disease	−0.635	[−1.80, 0.53]	0.590	−0.053	−1.076	0.283
Geriatric pain measure	−0.249	[−0.33, −0.17]	0.042	−0.346	−5.944	<.001***
Postural awareness	0.086	[−0.11, 0.28]	0.098	0.050	0.870	0.386
HPLP – Self-actualization	−0.350	[−0.93, 0.23]	0.292	−0.143	−1.199	0.232
HPLP – Health responsibility	0.940	[0.37, 1.51]	0.290	0.328	3.239	0.001***
HPLP – Exercise	−0.512	[−1.45, 0.43]	0.476	−0.094	−1.075	0.284
HPLP – Nutrition	−0.477	[−1.41, 0.46]	0.475	−0.092	−1.003	0.317
HPLP – Stress management	0.749	[−0.02, 1.52]	0.390	0.158	1.920	0.057
HPLP – Interpersonal support	0.698	[0.07, 1.32]	0.317	0.181	2.200	0.029*
Self-compassion	1.631	[−1.37, 4.63]	1.519	0.071	1.074	0.284
Self-efficacy	0.396	[0.27, 0.53]	0.066	0.340	6.017	<.001***

Dependent variable: WHOQOL-BREF total score; higher scores indicate better quality of life. Model 1 included demographic variables (gender, age, chronic disease) and geriatric pain. Model 2 added healthy lifestyle behaviors (HPLP subdimensions) and postural awareness. Model 3 additionally included psychological variables (self-efficacy and self-compassion).

HPLP = Health-Promoting Lifestyle Profile; B = unstandardized coefficient; SE = standard error; β = standardized coefficient; ΔR² = change in explained variance.

*p < .05, **p < .01, ***p < .001.

In Step 1, demographic variables and geriatric pain significantly predicted quality of life, F(4,174) = 17.9, p < 0.001, explaining 29.1% of the variance. Geriatric pain was a significant negative predictor (β = −0.502, p < 0.001).

In Step 2, healthy lifestyle behaviors and postural awareness were added, significantly increasing the explained variance to 51.9% (ΔR² = 0.228, p < 0.001). Health responsibility (β = 0.325, p = .004) and interpersonal support (β = 0.329, p < 0.001) were significant positive predictors, while pain remained significant.

In Step 3, self-efficacy and self-compassion further improved the model (R² = 0.614; ΔR² = 0.095, p < 0.001). In the final model, geriatric pain (β = −0.346, p < 0.001), self-efficacy (β = 0.340, p < 0.001), health responsibility (β = 0.328, p = 0.001), and interpersonal support (β = 0.181, p = 0.029) remained significant predictors. Self-compassion was not a significant predictor (β = 0.071, p = 0.284).

Overall, the final model explained 61.4% of the variance in health-related quality of life.

## Discussion

In this study, older women reported higher levels of geriatric pain and lower self-compassion scores compared to older men. After Bonferroni correction, the most robust gender differences involved total self-compassion and several of its subdimensions, total geriatric pain and selected pain subdimensions, the coping-with-obstacles subdimension of self-efficacy, the ease subdimension of postural awareness, and total quality of life and its environmental domain. Women scored lower on coping with obstacles and higher on postural ease, whereas men reported higher total quality-of-life and environmental-domain scores. Differences observed at the uncorrected level in behavioral initiation, behavioral completion, HPLP self-actualization, pain intensity, and the psychological quality-of-life domain did not remain statistically significant and should therefore be interpreted cautiously. Correlation analyses showed that self-compassion was positively associated with self-efficacy, postural awareness, health-promoting behaviors, and quality of life, and negatively associated with geriatric pain. Self-efficacy was positively correlated with health-promoting behaviors and quality of life, and negatively correlated with pain. The strongest observed association was a moderate positive correlation between health-promoting behaviors and quality of life. Regression analyses indicated that geriatric pain was statistically predicted by self-compassion and postural awareness. Self-efficacy was a significant predictor across all HPLP subdimensions and the total score, whereas self-compassion contributed significantly to all subdimensions except stress management. Similarly, postural awareness, geriatric pain, and gender significantly predicted several HPLP subdimensions. In the model predicting quality of life, geriatric pain was a significant negative predictor, while self-efficacy was a significant positive predictor; health responsibility and interpersonal support also contributed significantly to the model. To our knowledge, this study is among the first to examine self-compassion, self-efficacy, postural awareness, health-promoting behaviors, and quality of life simultaneously within a single biopsychosocial model in community-dwelling older adults.

Chronic and musculoskeletal pain conditions are reported to occur more frequently and with greater burden among women across the lifespan [50–52]. Studies conducted in Turkey similarly indicate higher pain prevalence and pain-related functional impairment among older women [53–55]. Consistent with this literature, the present study also found moderate levels of geriatric pain overall and significantly higher pain levels among women. Correlation analyses indicated that geriatric pain was negatively associated with self-compassion, self-efficacy, health-promoting lifestyle behaviors, and quality of life. In the regression model, self-compassion and postural awareness emerged as significant negative predictors of geriatric pain. This finding is consistent with studies suggesting that psychological and somatic awareness–based resources, such as self-compassion and postural awareness, may be associated with pain perception and pain coping processes [20,56]. In contrast, although self-efficacy approached statistical significance, it did not emerge as a significant predictor in the regression model. This may suggest that self-efficacy is related to pain outcomes indirectly —potentially via engagement in health-promoting behaviors and adaptive functional coping—rather than being directly associated with pain perception; this interpretation, however, is post hoc and cannot be tested within the present cross-sectional data.

The observed gender differences should also be interpreted cautiously within the Turkish sociocultural context. Previous studies among older adults in Turkey have reported associations of quality of life with gender, marital status, living arrangements, income, and perceived social support. These contextual factors may shape health experiences in later life; however, they do not explain the present differences in pain or self-compassion, and the underlying mechanisms require further cross-cultural investigation [57,58]

It is also important to distinguish postural awareness from related but conceptually distinct constructs. Postural awareness refers specifically to the conscious perception and monitoring of one’s body posture and alignment, whereas body awareness is a broader construct encompassing the perception of a wide range of bodily signals, and interoception refers more narrowly to the perception of internal physiological states (e.g., heartbeat, respiration, hunger). Although these constructs are interrelated, the present study assessed postural awareness specifically, and the findings should not be generalized to body awareness or interoception more broadly.

Regarding postural awareness, no significant gender differences were observed in total scores, consistent with previous literature [22,59]. Regression analysis indicated that the model predicting postural awareness was statistically significant; however, the explained variance was relatively low (R² = 0.048). Within this model, self-compassion emerged as a significant positive predictor of postural awareness, whereas self-efficacy was not a significant predictor. This finding aligns with studies suggesting that individuals with higher levels of self-compassion may demonstrate greater openness, acceptance, and awareness of bodily sensations [60,61]. Furthermore, correlation analyses revealed that postural awareness was positively associated with health-promoting lifestyle behaviors and quality of life and negatively associated with geriatric pain. These associations suggest that postural awareness may be related to broader somatic and behavioral processes; however, motor control, proprioception, and pain modulation were not directly assessed in the present study. Indeed, previous research has emphasized the role of body awareness and interoceptive processes in the regulation of pain perception and adaptation to chronic pain [22,62]. These findings are also consistent with the embodiment framework, which conceptualizes the body not merely as a passive reflection of emotional states but as an active regulatory system shaping cognitive and emotional processes [63,64]. Empirical studies have shown that slumped posture is associated with more negative thoughts and lower cognitive performance, whereas upright and open postures may facilitate emotional recovery and cognitive flexibility [65–68]. Accordingly, postural awareness may represent a multidimensional construct related to bodily, cognitive, and emotional processes, including self-compassion. Importantly, because this model explained only a small proportion of the variance, the psychological resources examined here account for little of the variability in postural awareness in this sample. These findings should therefore be interpreted with considerable caution, as postural awareness is likely shaped by additional factors—such as motor experience, physical activity history, and proprioceptive function—that were not assessed in the present study.

Hierarchical regression analyses in this study indicated that the stepwise model structure—demographic variables first, followed by geriatric pain, and finally psychological and body-related resources—provided a meaningful explanatory framework for HPLP. The relatively low variance explained by demographic variables suggests that behavioral engagement in older adults may be shaped more strongly by clinical burden and self-regulatory resources. When geriatric pain was added to the model, the explained variance increased substantially, particularly for nutrition and exercise. Pain negatively predicted self-actualization, health responsibility, exercise, nutrition, and the total HPLP score. This pattern is consistent with evidence showing that older adults with chronic musculoskeletal pain often report lower physical activity levels than their pain-free peers [69–71]. These findings are consistent with a close association between pain and health-promoting behaviors in older populations, although the direction of this association cannot be established here.

With the inclusion of psychosocial variables, self-efficacy emerged as a significant positive predictor across all HPLP subdimensions and the total score. This finding aligns closely with Social Cognitive Theory, which emphasizes the central role of self-efficacy in initiating, maintaining, and regulating health behaviors [72]. Recent meta-analytic evidence also demonstrates a moderate positive association between exercise self-efficacy and physical activity in older adults (r ≈ 0.41), with self-efficacy significantly predicting activity participation [73]. Thus, the consistent role of self-efficacy observed in this study is in line with theoretical and empirical evidence positioning it as an important correlate of health behaviors in healthy aging.

Self-compassion appeared to play a complementary role. It positively predicted self-actualization, health responsibility, exercise, nutrition (strongest effect), interpersonal support, and the total HPLP score, although it was not significant for stress management. These findings are consistent with meta-analyses reporting that self-compassion is positively associated with health-promoting behaviors such as healthy eating, exercise, and sleep [29,74,75]. The association may partly reflect the link between self-compassion and lower self-criticism and more adaptive emotional regulation, which in turn may relate to greater engagement in self-care behaviors. Nevertheless, meta-analytic associations between self-compassion and physical health or health-promoting behaviors are generally small to moderate and vary across health domains; accordingly, the present findings should not be generalized to all health behaviors or clinical outcomes [74].

Postural awareness also contributed significantly to several lifestyle domains, including self-actualization, health responsibility, exercise, stress management, and the total HPLP score. Its strongest association was observed for stress management, suggesting that the ability to perceive and regulate bodily signals may be associated with behavioral responses to stress. This interpretation is consistent with previous work showing that postural awareness is positively associated with mindfulness and body awareness, and negatively related to stress and clinical burden indicators [22]. Together, these findings highlight the possible role of body awareness as an embodied correlate of health behavior engagement in later life.

The hierarchical regression model predicting quality of life (WHOQOL-BREF total) demonstrated substantial explanatory power (Model 3: R² = 0.614), supporting a biopsychosocial interpretation of well-being in older adults. Geriatric pain remained a negative predictor across all steps of the model (β = −0.502 in Model 1; β = −0.346 in Model 3), suggesting that pain in later life functions not merely as a symptom but as a multidimensional burden associated with functioning, independence, and psychosocial well-being. When HPLP were added to the model, the explained variance increased markedly (ΔR² = 0.228). Because the HPLP subdimensions were moderately to strongly intercorrelated, their simultaneous inclusion may have reduced some individual coefficients through shared variance. Therefore, non-significant coefficients should be interpreted as limited unique contributions after adjustment for the other lifestyle domains, rather than as evidence of no association. In particular, health responsibility and interpersonal support emerged as significant positive contributors to quality of life, indicating that well-being in older adults is associated not only by health status but also with active engagement in health management and access to social resources. The addition of psychological variables further improved the model (ΔR² = 0.095), with self-efficacy emerging as a positive predictor of quality of life (β = 0.340). This finding aligns with Social Cognitive Theory, which posits that beliefs about personal control and capability facilitate coping with stressors and support self-care behaviors [72]. Previous research also suggests that self-efficacy may influence quality of life both directly and indirectly through functional capacity and health behaviors [76].

Interestingly, although self-compassion showed a moderate positive bivariate correlation with quality of life (ρ = 0.424), it did not remain a significant predictor once self-efficacy and the health-promoting behavior subdimensions were entered in the final model (β = 0.071, p = 0.284). This attenuation is consistent with substantial shared variance between self-compassion and the other resources in the model: self-compassion was moderately correlated with self-efficacy (ρ = 0.376) and was itself a significant predictor of health-promoting behaviors, including nutrition and the total behavior score. Its association with quality of life may therefore operate largely indirectly—through self-efficacy and engagement in health-promoting behaviors—rather than as an independent direct contribution. Meta-analytic evidence indicating that self-compassion is positively associated with health behaviors such as exercise and healthy eating supports this interpretation [29,75]. Thus, rather than functioning as a secondary factor, self-compassion may act as a complementary self-regulatory resource associated with behavioral engagement alongside self-efficacy. These remain hypotheses about indirect pathways that the present cross-sectional design cannot test directly; formal mediation models examining whether self-efficacy and health-promoting behaviors mediate the association between self-compassion and quality of life are needed to evaluate them in future longitudinal research.

Although several associations were statistically significant, their magnitudes were generally weak to moderate, and most individual standardized coefficients were modest. The geriatric pain and postural awareness models explained 14.1% and 4.8% of the variance, respectively, whereas the lifestyle behavior and quality-of-life models showed greater explanatory power. These findings therefore support a multicomponent interpretation rather than the use of any single psychological or body-awareness construct as a standalone clinical target. Moreover, because minimal clinically important differences and changes over time were not assessed, clinical significance cannot be inferred from statistical significance alone.

Taken together, pain, self-efficacy, self-compassion, and postural awareness may represent relevant components for future multicomponent geriatric rehabilitation research. Experimental posture studies provide a rationale for further investigation of body-awareness components, but they do not establish clinical effectiveness in older adults [65,77]. Similarly, the present cross-sectional findings cannot demonstrate that interventions targeting these constructs would improve health-promoting behaviors or quality of life. Longitudinal and controlled intervention studies are needed to determine whether such approaches produce clinically meaningful benefits.

Because of the cross-sectional design, the direction of the observed associations cannot be determined, and reverse or bidirectional relationships are equally plausible. Rather than self-compassion and self-efficacy contributing to lower pain and better quality of life, older adults who experience less pain and report better quality of life may in turn express higher levels of self-compassion and self-efficacy. Likewise, greater engagement in health-promoting behaviors may foster these psychological resources as much as the reverse. These alternative pathways cannot be ruled out within the present data and warrant examination in longitudinal and experimental designs. Accordingly, the term “predictor” used in the regression analyses refers to statistical prediction rather than a causal effect.

The findings should also be interpreted in light of several methodological limitations. The use of self-report measures within a single session may have introduced recall, social desirability, and common-method biases. Although Harman’s single-factor test did not identify a dominant general factor, this test does not completely rule out common-method variance.

Several potentially important confounders, including depression, anxiety, pain catastrophizing, socioeconomic status, cognitive function, functional capacity, physical performance, physical activity level, and detailed pain characteristics, were not comprehensively assessed. In addition, convenience sampling from healthcare facilities may have introduced selection bias by favoring older adults with greater healthcare access, health awareness, or preserved functional capacity. Consequently, the findings may not be fully generalizable to frail, socioeconomically disadvantaged, institutionalized, or severely functionally limited older adults. Future studies should use longitudinal, representative, and multi-method designs incorporating objective functional and physical activity assessments.

## Conclusion

In conclusion, higher self-efficacy was consistently associated with health-promoting behaviors and quality of life, whereas self-compassion and postural awareness showed domain-specific associations with pain and lifestyle behaviors. Geriatric pain remained negatively associated with quality of life. These findings support the consideration of pain burden and self-efficacy within a broader biopsychosocial assessment of community-dwelling older adults. Whether interventions targeting psychological or body-awareness resources can improve health behaviors or quality of life should be examined in longitudinal and controlled studies.

## Supporting information

S1 Table Complete correlation matrix including the HPLP and WHOQOL-BREF subdimensions. (DOCX)
