# Supplementary material for: Self-compassion and self-efficacy as biopsychosocial resources in healthy aging: Associations with pain, health behaviors, postural awareness, and quality of life
Source: PLoS One. 2026 Sep 16;21(9):e0356869. doi: 10.1371/journal.pone.0356869 (PMC13580965; doi:10.1371/journal.pone.0356869)
Supplement: Supplementary file 1 [file pone.0356869.s001.docx]

**S1 Table. Spearman’s rank correlations among total and subdimension scores**

| **Variable** |  | **Age** | **Self-Compassion Scale** | **Self-Efficacy Scale** | **Postural Awareness Scale** | **Geriatric Pain Measure** | **HPLP – Self-actualization** | **HPLP – Health responsibility** | **HPLP – Nutrition** | **HPLP – Stress management** | **HPLP – Interpersonal support** | **HPLP – Exercise** | **HPLP – Total** | **WHOQOL-BREF – Physical Health** | **WHOQOL-BREF – Psychological** | **WHOQOL-BREF – Social Relationships** | **WHOQOL-BREF – Environment** | **WHOQOL-BREF – Total Score** |
| --- | --- | --- | --- | --- | --- | --- | --- | --- | --- | --- | --- | --- | --- | --- | --- | --- | --- | --- |
| **Age** | ***p*** | — |  |  |  |  |  |  |  |  |  |  |  |  |  |  |  |  |
|  | ***ρ*** |  |  |  |  |  |  |  |  |  |  |  |  |  |  |  |  |  |
| **Self-Compassion Scale** | ***p*** | 0.405 | — |  |  |  |  |  |  |  |  |  |  |  |  |  |  |  |
|  | ***ρ*** | -0.063 |  |  |  |  |  |  |  |  |  |  |  |  |  |  |  |  |
| **Self-Efficacy Scale** | ***p*** | 0.027 | <0.001 | — |  |  |  |  |  |  |  |  |  |  |  |  |  |  |
|  | ***ρ*** | -0.165* | 0.376*** |  |  |  |  |  |  |  |  |  |  |  |  |  |  |  |
| **Postural Awareness Scale** | ***p*** | 0.092 | 0.004 | 0.586 | — |  |  |  |  |  |  |  |  |  |  |  |  |  |
|  | ***ρ*** | -0.126 | 0.215** | 0.041 |  |  |  |  |  |  |  |  |  |  |  |  |  |  |
| **Geriatric Pain Measure** | ***p*** | 0.898 | <0.001 | 0.033 | 0.001 | — |  |  |  |  |  |  |  |  |  |  |  |  |
|  | ***ρ*** | 0.010 | -0.267*** | -0.159* | -0.238** |  |  |  |  |  |  |  |  |  |  |  |  |  |
| **HPLP – Self-actualization** | ***p*** | 0.775 | <0.001 | <0.001 | <0.001 | <0.001 | — |  |  |  |  |  |  |  |  |  |  |  |
|  | ***ρ*** | -0.022 | 0.403*** | 0.308*** | 0.246*** | -0.336*** |  |  |  |  |  |  |  |  |  |  |  |  |
| **HPLP – Health responsibility** | ***p*** | 0.711 | <0.001 | <0.001 | <0.001 | <0.001 | <0.001 | — |  |  |  |  |  |  |  |  |  |  |
|  | ***ρ*** | 0.028 | 0.342*** | 0.261*** | 0.261*** | -0.361*** | 0.851*** |  |  |  |  |  |  |  |  |  |  |  |
| **HPLP – Nutrition** | ***p*** | 0.737 | <0.001 | <0.001 | 0.020 | <0.001 | <0.001 | <0.001 | — |  |  |  |  |  |  |  |  |  |
|  | ***ρ*** | -0.025 | 0.504*** | 0.340*** | 0.174* | -0.385*** | 0.650*** | 0.653*** |  |  |  |  |  |  |  |  |  |  |
| **HPLP – Stress management** | ***p*** | 0.385 | <0.001 | <0.001 | <0.001 | 0.004 | <0.001 | <0.001 | <0.001 | — |  |  |  |  |  |  |  |  |
|  | ***ρ*** | -0.065 | 0.287*** | 0.247*** | 0.394*** | -0.214** | 0.672*** | 0.631*** | 0.621*** |  |  |  |  |  |  |  |  |  |
| **HPLP – Interpersonal support** | ***p*** | 0.134 | <0.001 | <0.001 | 0.068 | 0.007 | <0.001 | <0.001 | <0.001 | <0.001 | — |  |  |  |  |  |  |  |
|  | ***ρ*** | -0.113 | 0.344*** | 0.447*** | 0.137 | -0.201** | 0.705*** | 0.634*** | 0.544*** | 0.623*** |  |  |  |  |  |  |  |  |
| **HPLP – Exercise** | ***p*** | 0.887 | <0.001 | 0.005 | <0.001 | <0.001 | <0.001 | <0.001 | <0.001 | <0.001 | <0.001 | — |  |  |  |  |  |  |
|  | ***ρ*** | -0.011 | 0.277*** | 0.207** | 0.267*** | -0.374*** | 0.746*** | 0.715*** | 0.666*** | 0.594*** | 0.587*** |  |  |  |  |  |  |  |
| **HPLP – Total** | ***p*** | 0.568 | <0.001 | <0.001 | <0.001 | <0.001 | <0.001 | <0.001 | <0.001 | <0.001 | <0.001 | <0.001 | — |  |  |  |  |  |
|  | ***ρ*** | -0.043 | 0.411*** | 0.348*** | 0.272*** | -0.351*** | 0.943*** | 0.901*** | 0.758*** | 0.781*** | 0.812*** | 0.814*** |  |  |  |  |  |  |
| **WHOQOL-BREF– Physical health** | ***p*** | 0.175 | 0.003 | <0.001 | <0.001 | <0.001 | <0.001 | <0.001 | <0.001 | <0.001 | <0.001 | <0.001 | <0.001 | — |  |  |  |  |
|  | ***ρ*** | -0.102 | 0.224** | 0.364*** | 0.281*** | -0.713*** | 0.359*** | 0.372*** | 0.310*** | 0.294*** | 0.349*** | 0.397*** | 0.407*** |  |  |  |  |  |
| **WHOQOL-BREF– Psychological domain** | ***p*** | 0.958 | <0.001 | <0.001 | 0.009 | <0.001 | <0.001 | <0.001 | <0.001 | <0.001 | <0.001 | <0.001 | <0.001 | <0.001 | — |  |  |  |
|  | ***ρ*** | 0.004 | 0.553*** | 0.518*** | 0.194** | -0.422*** | 0.496*** | 0.482*** | 0.503*** | 0.466*** | 0.456*** | 0.431*** | 0.541*** | 0.618*** |  |  |  |  |
| **WHOQOL-BREF– Social relationships** | ***p*** | 0.894 | 0.003 | <0.001 | 0.346 | 0.002 | <0.001 | <0.001 | <0.001 | <0.001 | <0.001 | <0.001 | <0.001 | <0.001 | <0.001 | — |  |  |
|  | ***ρ*** | 0.010 | 0.221** | 0.356*** | 0.071 | -0.226** | 0.416*** | 0.510*** | 0.284*** | 0.346*** | 0.505*** | 0.327*** | 0.469*** | 0.378*** | 0.515*** |  |  |  |
| **WHOQOL-BREF – Environmental domain** | ***p*** | 0.873 | <0.001 | <0.001 | 0.013 | <0.001 | <0.001 | <0.001 | <0.001 | <0.001 | <0.001 | <0.001 | <0.001 | <0.001 | <0.001 | <0.001 | — |  |
|  | ***ρ*** | 0.012 | 0.434*** | 0.501*** | 0.186* | -0.366*** | 0.444*** | 0.519*** | 0.399*** | 0.443*** | 0.371*** | 0.265*** | 0.482*** | 0.558*** | 0.719*** | 0.557*** |  |  |
| **WHOQOL-BREF – Total** | ***p*** | 0.790 | <0.001 | <0.001 | 0.002 | <0.001 | <0.001 | <0.001 | <0.001 | <0.001 | <0.001 | <0.001 | <0.001 | <0.001 | <0.001 | <0.001 | <0.001 | — |
|  | ***ρ*** | -0.020 | 0.424*** | 0.497*** | 0.227** | -0.529*** | 0.520*** | 0.576*** | 0.444*** | 0.460*** | 0.500*** | 0.434*** | 0.575*** | 0.793*** | 0.860*** | 0.732*** | 0.846*** |  |

* p < 0.05, ** p < 0.01, *** p < 0.001. SCS = Self-Compassion Scale; HPLP = Health-Promoting Lifestyle Profile; WHOQOL-BREF = World Health Organization Quality of Life
